# Strategies for regeneration of components of nervous system: scaffolds, cells and biomolecules

**DOI:** 10.1093/rb/rbu017

**Published:** 2015-01-13

**Authors:** Lingling Tian, Molamma P. Prabhakaran, Seeram Ramakrishna

**Affiliations:** ^1^Mechanical Engineering, Faculty of Engineering, National University of Singapore, 2 Engineering Drive 3, Singapore 117576 and ^2^Nanoscience and Nanotechnology Initiative, National University of Singapore, 2 Engineering Drive 3, Singapore 117576

**Keywords:** electrospinning, nerve tissue engineering, nanofibers, functionalized scaffolds

## Abstract

Nerve diseases including acute injury such as peripheral nerve injury (PNI), spinal cord injury (SCI) and traumatic brain injury (TBI), and chronic disease like neurodegeneration disease can cause various function disorders of nervous system, such as those relating to memory and voluntary movement. These nerve diseases produce great burden for individual families and the society, for which a lot of efforts have been made. Axonal pathways represent a unidirectional and aligned architecture allowing systematic axonal development within the tissue. Following a traumatic injury, the intricate architecture suffers disruption leading to inhibition of growth and loss of guidance. Due to limited capacity of the body to regenerate axonal pathways, it is desirable to have biomimetic approach that has the capacity to graft a bridge across the lesion while providing optimal mechanical and biochemical cues for tissue regeneration. And for central nervous system injury, one more extra precondition is compulsory: creating a less inhibitory surrounding for axonal growth. Electrospinning is a cost-effective and straightforward technique to fabricate extracellular matrix (ECM)-like nanofibrous structures, with various fibrous forms such as random fibers, aligned fibers, 3D fibrous scaffold and core-shell fibers from a variety of polymers. The diversity and versatility of electrospinning technique, together with functionalizing cues such as neurotrophins, ECM-based proteins and conductive polymers, have gained considerable success for the nerve tissue applications. We are convinced that in the future the stem cell therapy with the support of functionalized electrospun nerve scaffolds could be a promising therapy to cure nerve diseases.

Mechanical, thermal, chemical or ischemic factors can lead to damage of the nervous system and impair system functions like memory, cognition, language and voluntary movement [1], which are extremely important for individual lives. The two most severe nerve injuries attracting the most attention from the society are spinal cord injury (SCI) and traumatic brain injury (TBI) because of the huge numbers of affected populations, the severe situation after the injuries and the enormous financial cost. SCI causes loss of neurons and axons, which might result in motor and sensory function impairments, and even death or permanent disability. Approximately 1.2 million people in the USA are paralyzed due to SCI [2], and it is estimated that 273 000 persons suffered from SCI in the USA (year 2013). From 2010, 36.5% of SCI has been caused by motor vehicle crash. And according to World Health Organization, 1.24 million people die each year on the world’s roads. For USA, this number is 35 490, and the estimated road traffic death rate is 11.4/100 000 population. For China, the estimated number of road traffic deaths is 275 983, and the estimated road traffic death rate is 20.5/100 000 population (year 2010) (http://apps.who.int/gho/data/node.main.A997). From these facts, we are convinced that a huge number of population suffered from SCI after road accidents in the whole world. The health care and living expenses that directly attributed to SCI are huge. For example, for high tetraplegia, the first year cost is estimated to be $ 1 044 197 and each subsequent year cost is estimated to be $ 181 238 (https://www.nscisc.uab.edu/PublicDocuments/fact_figures_docs/Facts%202012%20Feb%20Final.pdf). That will be a huge burden for individual families. According to ‘A 2013 Profile of Persons with Traumatic Brain Injury who Received Inpatient Rehabilitation’ from National Data and Statistical Center, Craig Hospital Englewood, Co, as of December, 2012, the database contained information on 11 772 persons with TBI, and the average age is 40.34 years (http://www.msktc.org/lib/docs/Data_Sheets_/2013_TBIMS_National_Database_Update.pdf). Most of them suffered from TBI at this active and young age, which causes great inconvenience for their future lives. In the USA, it is estimated that 1.4 million people undergo TBI while more than 5 million people are suffering from disabilities resulting from TBI at a cost of $ 56 billion a year (based on year 1995) [3]. Additionally, TBI will also trigger neurodegenerative disease [4].

Peripheral nerve injury (PNI) has attracted public attention because of the huge society and economic burden it has caused. No statistical data for PNI for recent years are found; however, we still can sense the severity of the peripheral injury from the following data. Economically speaking, nerve injuries cost $ 150 billion annually in the USA [5]. PNIs caused 8.5 million restricted activity days and 5 million disability days in the year 2004 [6, 7], and over 200 thousand procedures were performed to repair PNI annually in the USA [8].

The figures related to nerve injuries are shocking, which motivates the scientists to design a way to improve the current therapy and even help the regeneration of the injured nerve. As we know, nervous system is one of the most complex systems in human body, and thorough understanding of the organization, the cellular components and the anatomy of the nervous system is the first and essential step for treating the nervous injuries.

## Physiology of the Nervous System

### Organization of the nervous system

The vertebrate nervous system consists of two main parts: the central nervous system (CNS) and the peripheral nervous system (PNS). The CNS functions as a carrier and interpreter of signals, as well as a generator of excitatory stimuli to the PNS. The five main components of the CNS are brain, spinal cord, optic, olfactory and auditory systems [8], and however brain and spinal cord are the most representative parts. CNS neurons are intrinsically capable of regeneration of damaged axons to some extent [9], but the capability is very limited [10, 11]. The connection between the CNS and peripheral structures is the PNS [12], through which sensory and excitatory signals are transmitted in both directions (from or to the spinal column). In addition, the PNS is responsible for innervating muscle tissue. The PNS, a collection of nerves, sensory receptors and ganglia outside the CNS, is one of the largest and most complex structures in the body, and most components of which are produced at various stages of the embryonic development.

### Cellular components of the nervous system

Neurons and neuroglia are two main cell categories in the nervous system. Neurons are composed of soma, axons and dendrites. Ganglia are clusters of sensory nerve soma. Dendrites propagate electrical signals received from other neural cells to soma, while axon typically conducts electrical impulses away from the neuron’s cell body. Neuroglia, are support cells, which refer to Schwann cells (PNS), astrocytes and oligodendrocytes (CNS). Neuroglia are more plentiful than neurons, and they are capable to divide [8].

Biologically, Schwann cells (SCs) is to myelinate and ensheath nerve fibers in the PNS. In addition, SCs secrete neurotrophins and produce extracellular matrix (ECM) molecules [13], which facilitates nerve regeneration. Regenerative axons migrated aligned with SCs, and nerve gaps were bridged [14]. And in contrast, obstructed migration of SCs might hinder the regeneration of the axons, leading to recovery failure [15]. The absence of SCs in the CNS is the biggest barrier for regeneration [16].

Besides neurons and neuroglia, other cells, such as neural stem cells (NSCs) and olfactory ensheathing glial cells (OEGs), also played important roles in the nervous system. NSCs is able to self-renew, as well as generate main neuronal and glial cells [17]. Human NSCs are able to differentiate into the three fundamental neuronal lineages (neurons, astrocytes and oligodendrocytes), as well as achieving full neuronal maturation [18], but which will be affected by the environments, such as growth factors [19], dynamic forces [20] or the presence of another cell type [21]. Transplantation of NSCs into the adult CNS could not only enhance functional recovery [22], but also remodel the injured tissue [23] and increase the tissue plasticity [24]. OEGs, particularly existing in the CNS, keep proliferating throughout the life, supporting and guiding the growth of newly formed axons which has shown great potential in CNS neural repair [25]. The CNS glial scars cannot block the OEG migration and OEGs are able to enter both gray and white matter, potentially attracting regenerating axon [25]. In the past two decades, it has been extensively proved that OEGs are effective in promoting axonal regeneration in the injured adult mammalian CNS [25–35].

### Anatomy of the PNS and CNS

Bound motor and sensory axons are supported by the surrounding tissue and well vascularized by capillaries and vessels [36], and that is an anatomically defined structure—a peripheral nerve. There are three different kinds of connective tissues located in different places, namely endoneurium, perineurium and epineurium. Inside the fascicles, endoneurium holds neurons and blood vessels in place; perineurium, a collection of flattened cells and collagen fibers, is to surround and hold together each fascicle; epineurium covers and holds together the nerve fascicles.

The spinal cord comprises white and gray matter. The butterfly-shaped gray matter, consisting of numerous neuronal cell bodies, dendrites, few myelinated, unmyelinated axons, glial cells and capillaries, is located in the center. The white matter, consisting mainly of glial cells and myelinated axons, surrounds and protects the gray matter.

In terms of embryonic development, the brain can be divided into three main parts: the forebrain, midbrain and hindbrain. The forebrain comprises cerebrum, thalamus, hypothalamus and pineal gland among other features. The midbrain comprises a portion of the brainstem. The hindbrain comprises the remaining brainstem, and the cerebellum and pons. Brainstem connects the brain to the spinal cord, and it is the most inferior portion of the brain.

In summary, the physiology of the nervous system is shown as in Table 1.

**Table 1.** Physiology of the nervous system

Nervous system			
Organization	CNS		PNS
	Brain		Cranial nerves arising from the brain
	Spinal cord		Spinal nerves arising from the spinal cord
	Optic		Sensory nerve cell bodies
	Olfactory		
	Auditory		
Cellular components	Neuron		Neuron
	Neuroglia (Astrocyte oligodendrocyte)		Neuroglia (SCs)
Anatomy	Brain	Spinal cord	
	Forebrain	Gray matter	Endoneurium
	Midbrain	White matter	Perineurium
	Hindbrain		Epineurium

## Trauma of Nervous System, Current Standard of Care and Challenges of Tissue Regeneration

No matter the injury types of nerve (acute or chronic), the common feature is the loss of neurons, or additionally the loss of supporting neural cells [37]. But therapeutic intrusion has to be specific as tissue organization and basic cell types of the peripheral nerves, brain, spinal cord vary significantly and different cells react to injury in different ways.

### PNI and the treatments

After injury, the functional recovery can be gained in the PNS [6] because of the presence of SCs, which are able to supply nutrient support, guide and myelinate regenerating axons, and provide growth-promoting molecules and growth factors [38]. The detailed process is described as following: when nerve injury happens in the PNS, the distal end of axon degrades after being cut off from the central cell body; SCs and microphages take part in the process of Wallerian degeneration, which clear away the resulting debris of the axon; the SCs act positively in three forms: proliferation, formation of ‘bands of Bungner’ and secretion of conductive factors [39]; and at the same time, the injured neuron is ready for regeneration. The regenerating axon of the distal nerve segment develops toward the original target, and the speed is in the range of 1–3 mm per day [40].

For curing PNI with small gaps, surgical reconnection is commonly used. But sometimes mismatching of nerve fascicles or time delay has a negative influence on recovery of nerve function [41]. When the nerve gap is longer, and primary repair cannot be achieved promptly, nerve autografts are considered as the gold standard. But the disadvantages do exist including significant donor site morbidity, insufficient donor nerve availability and the need for extra operative procedure [42]. Allografts and xenografts [43–45] are also considered as alternatives, but the resultant immune rejection limits the applications. Full functional recovery seems impossible although a lot of efforts have been made [46]. There are various undesirable consequences of partial recovery from injury of peripheral nerves, such as numbness, loss of sensory function or mobility, and likelihood of developing chronic pain and permanent disability. The situation has prompted the development of novel nerve scaffolds.

### SCI and the treatments

CNS axons lack the ability to regenerate by themselves because of the inhibitory surroundings, while PNS does. After SCI, the spinal cord might be smashed by a fractured vertebra, and the possible consequences might be injured neural cells, severed axonal networks, and disrupted blood vessels. The instant consequence is swollen spinal cord, which further disturbs blood flow. After these primary responds after SCI, the area of cell death and tissue damage are expanded. Edema and ischemic cell death continue, which triggers disastrous inflammation, accompanied with infiltration of cells through the compromised blood–spinal cord barrier. The astrocytes and microglia response, and a growth-inhibitory factors contained glial scar forms. Additionally, some glycoproteins existing in the myelin are major growth-inhibitory factors [47]. Some proteins expressed by oligodendrocytes, such as tenascin R and myelin-associated glycoprotein (MAG) [48, 49], hinder the outgrowth of axons. Additionally, local astrocytes are triggered and contribute to forming a scar, which impedes the axonal outgrowth significantly [50, 51]. In the CNS, however, endogenous components do not support axonal elongation. And regeneration may occur with supporting substrates [52], but the recovery effect is limited. When CNS injury happens, permanent functional damage is the general consequence following the progressive injury process.

There are great limitations about the current therapies for spinal cord repair and regeneration. Initial damages result in a quick happening of acute primary injury events, and the purpose of existing treatments is to prevent the extent of the secondary injury, which frequently involves the application of high doses of steroids [53]. But unfortunately even for this consideration, rare choices for clinical applications exist.

### TBI, neurodegenerative disease and the treatments

Brain is a complicated structure which includes delicately connected neurons that are supported by the ECM [54]. The brain is a part of CNS, like spinal cord. So they share similar properties, such like glial cell types and the presence of blood–brain barrier which limits diffusion of therapeutics using conventional delivery strategies [55]. When TBI and degenerative disease happen, as in the spinal cord, secondary injury cascades cause further damage and cell death; growth inhibitory molecules block regenerating axons, and CNS neurons cannot proliferate; finally, injury and neurodegenerative disease can cause imbalances of neurotransmitters, resulting in severe loss of function. As a unique part of human body, the brain comprises unique components, many of which have particular neurons to play specialized roles. The objective of injury, disorder and disease can selectively be the particular parts of the brain. For example, the target region of Parkinson’s disease (neurodegenerative disease) is the basal ganglia, and the neuron’s function of producing the neurotransmitter dopamine will be destroyed.

Similar to SCI, minimizing the further injury after TBI is the treatment purpose and strategy due to the irreversible initial brain damage [56]. Adequate oxygen supply, blood flow and pressure are three most crucial parameters for immediate treatment, by controlling which the organ viability can be maintained. For the case involving more severe injuries, surgery is needed to remove hematomas and repair contusions [57].

**Figure 1.**
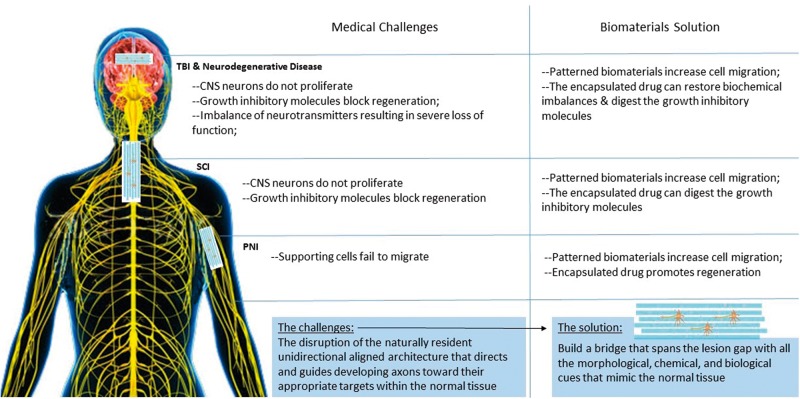
The medical challenges and biomaterials solution for nervous injury and neurodegenerative disease.

In summary, axonal pathways represent a unidirectional and aligned architecture allowing systematic axonal development within the nerve tissue, and following PNI, SCI, TBI or neurodegenerative disease, the intricate architecture suffers disruption leading to the inhibition of growth and loss of guidance [58]. Due to limited capacity of the body to regenerate axonal pathways, it is desirable to have a biomimetic approach that has the capacity to graft a bridge across the lesion while providing optimal morphological, chemical and biological cues for nerve tissue restoration. The CNS axons are unable to regenerate due to the inhibitory tissue surrounding after injury. Hence one extra strategy is needed for SCI and TBI: breaking the barrier created by the inhibitory environment of the injury gap. The medical challenges and biomaterials solution for nervous injury and neurodegenerative disease is shown as Fig. 1.

## Commercialized Guidance Conduits and Wraps for PNI

Available nerve implants in the markets are mainly for PNIs, and they can be classified into three categories: peripheral nerve allografts, porcine material-based implants and biomaterial peripheral nerve scaffolds. The details are listed as in Table 2.

**Table 2.** Commercialized guidance conduits and wraps for PNI

Supplier	Product	Form	Materials
AxoGen	Avance Nerve Graft		Allograft
	AxoGuard Nerve Connector	Conduit	Porcine material
	AxoGuard Nerve Protector	Wrap	Porcine material
Stryker	NeuroMend	Wrap	Collagen
	NeuroMatrix	Conduit	Collagen
	NeuroFlex	Conduit	Collagen
Integra	NeuraGen	Conduit	Collagen
	NeuraWrap	Wrap	Collagen
Polyganics	Neurolac	Conduit	PLA-CL
Synovis Micro	NeuroTube	Conduit	PGA

### Allografts and procine material-based devices

Avance® Nerve Graft is an acellular ECM obtained from donated human peripheral nerve tissue. Seven patients were treated surgically using this allograft, and adequate sensation in nerve defects ranging from 0.5 to 3 cm without infection or rejection was obtained [59]. AxoGuard® Nerve Connector is a conduit, which is used to align and connect nerves with less than a 5-mm gap between the severed nerve ends. AxoGuard®Nerve Protector is a wrap, which is designed to protect and isolate the nerve during the healing process after surgery. The patient’s own cells incorporate into the ECM to remodel and form a tissue similar to the nerve epineurium.

### Natural polymer-based devices

For biomaterial nerve scaffolds, silicone was the earliest synthetic nerve conduit to be used, since it is inert, readily available and, to an extent, elastic [60]. But in the clinical setting, many patients complained of irritation at the site of surgery, which required subsequent removal. Therefore, the method has become clinically unpopular [61]. Polytetrafluoroethylene (PTFE) was introduced as a polymer for spanning gaps of up to 40 mm, but, over time, it was found to compress the nerve, leading to altered nerve regeneration as well as causing irritation to the patient. Collagen is an abundant structural protein of various connective tissues in animals. And it is the most prevalent component of the ECM. Due to its high biocompatibility, collagen has been the most popular natural polymer for fabricating tissue engineered scaffolds.

With time, nerve conduits fabricated from biocompatible materials gain success, and FDA has approved several devices for peripheral nerve, including Type I collagen based, Poly _D,L_lactide-co-ε-carprolactone-based and PGA-based devices.

Collagen is found naturally as a triple-helical protein in mammals [62]. It is the most abundant protein in the human body and the main component of the ECM [63]. As the main component of connective tissue, it is the most abundant protein in mammals, making up from 25% to 35% of the whole-body protein content. In humans, collagen comprises one-third of the total protein, accounts for three quarters of the dry weight of skin [64]. In nervous tissue regeneration, collagen has been used in scaffolds [65], magnetically aligned fibrils [39], gels [66] and cell-delivery vehicles [67]. Type I collagen and Type IV collagen remain the most commonly used nerve conduit, with Type I collagen being more biocompatible [68]. Type I collagen-based nerve conduits contain the following.

NeuroMend, Type I Collagen Nerve Wrap, is resorbable and semipermeable. It can unroll and self-curl to best match the dimensions of the injured nerve. The wrap is to protect the lesion part by preventing the formation of neuromas [69]. NeuroMatrix, is a tubular matrix designed to create a protective environment for axonal growth across a nerve gap. Neuro*flex*, a flexible Type 1 Collagen Conduit, is designed with the purpose of protecting the axonal growth across a nerve gap, of which the maximum length is 2.5 cm.

NeuraGen® Nerve Guide, a type I collagen tube designed to be an interface between the nerve and the surrounding tissue and to create a conduit for axonal growth across a gap. One year follow-up of 12 patients with nerve repair procedures by NeuraGen showed that, four obtained excellent results, five achieved good sensibility [70]. The level of functional recovery achieved with this nerve guide is equivalent to direct suture repair (animal trials) and is stable over 3–4 years [71]. NeuraWrap™ Nerve Protector is designed to provide a protection layer between the nerve and the surrounding tissue. The resilience of the collagen conduit allows NeuraWrap™ to recover and maintain closure once the device is placed around the nerve.

### Synthetic polymer-based devices

Neurolac® and Neurolac® TW are nerve conduits made from PLACL. It is promising to reconstruct a peripheral nerve discontinuity up to 20 mm in patients. Neurolac® TW is the thin-wall version of Neurolac®, it has a wall thickness that is 40% less than the Neurolac® conduits. Neurolac® was used to recover the sensory nerve function after traumatic peripheral nerve lesions, and 30 patients with 34 nerve lesions were included in this trial; recovery of sensibility was satisfactory, and the Neurolac nerve guide is suitable for the repair of transected nerves in the hand [72].

The GEM NeuroTube is made of polyglycolic acid (PGA) via weaving techniques. It is a absorbable tube, which aims for one-time use in patients with an injury to peripheral nerve gap ≥8 mm, but ≤3 cm. NeuroTube has been used to bridge a 3-cm nerve defects in two patients, and 2 years after reconstruction, the two point discrimination of thumb, index and middle finger were recovered with good localization for each patient [73].

**Figure 2.**
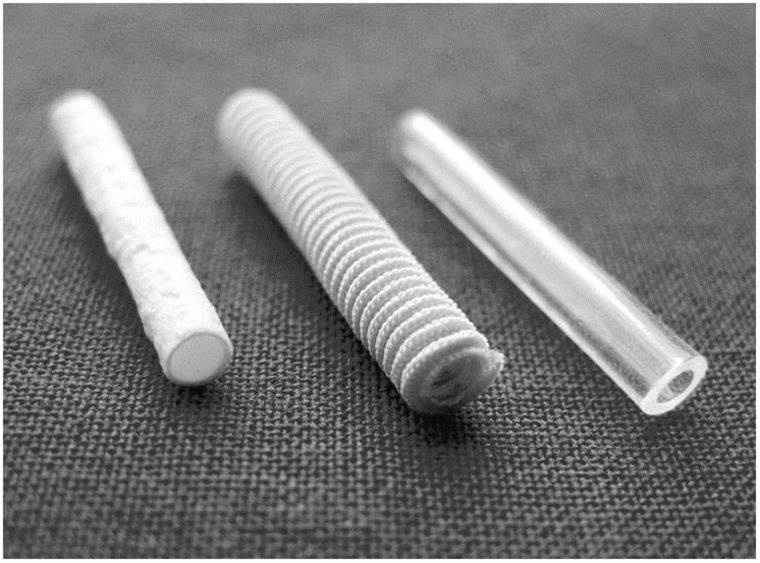
Nerve conduit: (**a**) Collagen I NeuraGen; (**b**) PGA NeuroTube; (**c**) PLACL Neurolac [70].

Currently, the nerve implants are available mainly in two forms: conduit and wrap, and they have gained success in the recovery of nerve injuries to some extent. The three main nerve conduits are shown as Fig. 2.

## Biomaterial and Electrospun Scaffolds Applied in Nerve TE

Presently, the forms of biomaterial nerve scaffolds mainly include: hydrogel, self-assembly peptide, nanofibers and their combinations. Hydrogel is a highly absorbent solid network of either natural or synthetic polymers, and it can be applied in tissue engineering, such as wound dressing [74], drug release [75], cell encapsulation [76], artificial organs [77] and tissue engineering [78]. For example, thermoresponsive xyloglucan hydrogel scaffolds functionalized through the immobilization of poly-d-lysine were fabricated, and it could support the differentiation of primary cortical neurons [79]. Another technique which has been applied in fabrication of nerve tissue scaffold is peptide self-assembling. Oligopeptides consisting of only natural amino acids dissolves in salt-free aqueous solutions, self-assembles into gels spontaneously. And nanofibers will be formed immediately when the peptide solution is exposed to salt solutions [80–84]. In this review, we focus on nanofibers fabricated by electrospinning method, and it will be introduced in detail.

**Figure 3.**
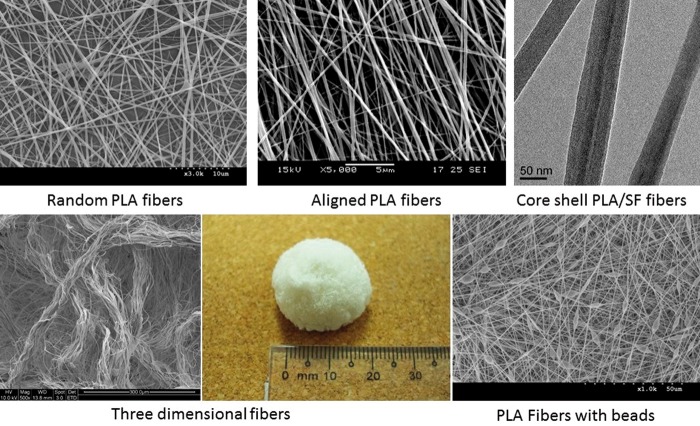
Diverse fibrous forms produced by electrospinning. (Contribution from Center for Nanofibers & Nanotechnology at NUS)

Nanoarchitectures have attracted much attention due to that materials in nanoscale can show unique properties such as the extraordinarily large surface to volume ratio. Nanofibers, as one form of nanoarchitectures which can resemble ECM structure closely, have been explored for a variety of tissue engineering application. The main ways to fabricate nanofibers consist of phase separation, self-assembly and electrospinning. Among these techniques, electrospinning is a cost-effective and efficient approach for making polymeric nanofibers from variety of polymers in diverse forms such as random fibers, aligned fibers, 3D fibers, fibrous conduits and core-shell fibers. Figure 3 shows the different fibrous forms produced by electrospinning method.

The general consequence of nerve disease or disorder is the disruption of the unidirectional architecture, and bridging the lesion part should be the first step. A supportive structure mimicking the natural nerve structure should be developed, and the key considerations include but not limited to: ensuring the certain porosity that allows cells’ migration. The further consideration should focus on mimicking the biological property of the native environment, like the proteins or signaling factors. In this regard, the biomaterial structure also should act as a drug carrier and be able to deliver drugs in a controlled manner to aid in the attachment, proliferation and viability of the cells [85], which is extremely important for SCI since the natural environment of SCI site provide inhibitory factors rather than supportive factors for nerve regenerations. Biocompatible materials with favorable morphological structures and biological properties which are able to guide regeneration axons could be an ideal bridging structure for injured nerve.

Electrospinning technique satisfies all the requirements to build such an ideal bridging prosthesis, and it requires a relatively simple setup and can be applied to a variety of polymers, including both synthetic and natural polymers.

Synthetic polymers like poly (glycolic acid) (PGA), poly (l-lactic acid) (PLLA) and poly (caprolactone) (PCL) and their copolymers have been extensively used to produce nanofibers via electrospinning method for nerve tissue applications. Besides, natural materials have a great potential to be more suitable for tissue engineering as their mechanical strength, physical properties and biomolecular recognition may be similar to native soft tissues [86]. Collagen, gelatin, laminin and chitosan have been explored to develop electrospun nanofibers for neural tissue engineering [87–89]. And besides, silk fibroin is one of two proteins excreted by *Bombyx mori* silkworms during cocoon production, and it has received significant attention as a versatile natural polymer due to its high strength-to-weight ratio and slow degradation [90].

The electrospun nanofibrous nerve scaffolds are sorted into four classes: randomly orientated nanofibers, aligned nanofibers, 3D nanofibers and functionalized nanofibers, and the details are shown as in Table 3.

**Table 3.** Electrospun polymers used for nerve tissue engineering

Polymer	CopolymerSecond Polymer	Fiber forms	Molecules	Applications	In vivo	In vitro	Year	References
PLA		Random		Nerve tissue		NSC	2004	[91]
PLA	PANi	Random		Nerve tissue		Rat C 17.2 (NSC)	2011	[92]
PLA		Aligned		Nerve regeneration		Embryonic stage nine (E9);chick DRG; rat SCs	2009	[93]
PLA		Aligned		Nerve regeneration		DRG explants	2007	[94]
PLA		Aligned	Laminin; bFGF	Nerve tissue		Rat DRGs; human dermal fibroblasts	2007	[95]
PLA	Gelatin		Retinoic acid (RA); purmorphamine	PNI		Neuronal stem cells (to motor neurons)	2014	[96]
PCL		Random	GRGDS (part of fibronectin, laminin, and other ECM molecules)	PNI		SCs	2011	[97]
PCL		Random		PNI			2008	[98]
PCL	Collagen	Random		Nerve implants		DRG explants; SCs	2007	[41]
PCL	Collagen I	Aligned		CNS		Astrocyte	2009	[99]
PCL	Gelatin	Aligned		Nerve regeneration		Human MSC; PC12	2014	[100]
PCL		Aligned		Nerve injury repair		Embryonic stem cells (ES)	2009	[101]
PCL		3D	Ethylenediamine (ED)	Nerve regeneration		NSCs, derived from rat brain	2008	[102]
PCL	PLGA	3D (tube)		Sciatic nerve	Rat		2008	[103]
PCL		3D		PNI		Embryonic chicken DRG	2014	[104]
PLGA		Aligned		Nerve tissue		Human nerve cells	2013	[105]
PLGA		3D, aligned		SCI	Rat	A-172, derived from human brain	2014	[106]
PLGA	SF	3D (conduit)		PNI	SD Rat		2012	[107]
PLGA	SF; Collagen	Random		Nerve tissue		SCs	2011	[108]
PLGA	Ppy (coating)	Aligned				PC12	2009	[109]
PLGA		Aligned; coaxial	NGF	PNI	Rat		2012	[110]
PLA-CL		Core-shell	Laminin	PNI		SCs	2014	[111]
PLA-CL	SF	Aligned		Nerve tissue		SCs	2013	[112]
PLA-CL	Collagen I; Collagen III	Aligned		PNI		C17.2	2012	[113]
PLA-CL	PAni	Random		Nerve tissue		PC12	2012	[114]
PLA-CL	SF	Core-shell	NGF	PNI	Rat		2013	[115]
Polydioxanone		Aligned; random		SCI		Rat DRGs; astrocytes	2007	[85]
PHBV	Chitosan(cross linker)	3D (conduit)		Sciatic nerve	Rat	SCs	2013	[116]
PHBV/PHB	Collagen	Aligned		Nerve tissue		SCs	2013	[117]
PCL-PEG block copolymer		Aligned	NGF (conjugated)			MSCs (neuronal differentiation)	2010	[118]
Methyl methacrylate-Acrylic acid (PMMAAA)	Collagen (immobilized)	Random				NSCs	2008	[119]
SF		Random		PNI		SCs	2012	[120]
SF		Aligned	Brain derived neurotrophic factor (BDNF); Ciliaryneurotrophic factor (CNTF)	CNI		Rat retinal ganglion cell (RGC)	2011	[121]
Chitosan	PVA	Random	NGF	Nerve tissue		Human glioblastoma-astrocytoma (U373-MG); human neuroblastoma (SKNMC)	2011	[122]
Chitosan		3D	CYIGSR (region of laminin-1)	Sciatic nerve		SD rats	2008	[123]
Poly(propylene carbonate) (PPC)		Aligned		PNI		SD rat DRGs; SCs	2011	[124]
Collagen		Random	Neurotrophin-3 (NT-3); chondroitinase ABC (ChABC)	SCI (CNS)		DRG	2012	[125]

### Randomly orientated nanofibers

Initially, randomly orientated nanofibers were electrospun and applied into tissue engineering applications due to its high similarity to ECM. Pure synthetic or natural polymers were electrospun. PLA nanofibers with interconnected pores were fabricated, and it resembled the natural extracellular matrix structure in human body. The results of *in vitro* cell culture study indicate that the nanofibers scaffold not only supports NSC differentiation and neurites outgrowth, but also promotes NSC adhesion [91]. Silk fibroin (SF) was fabricated, and SCs were cultured on these scaffolds. The findings indicate that regenerated electrospun SF nanofibers can promote SC adhesion, growth and proliferation, and have excellent biocompatibility [120].

To combine the advantages of both synthetic polymer (good mechanical properties, controllable degradation rate, etc.) and natural polymer (good biocompatibility), many attempts have been made to improve the property of electrospun nerve scaffolds. PLGA–SF–collagen nanofibers were fabricated through blending electrospinning, and SCs were seeded on the scaffold. The results confirmed that PLGA–SF–collagen scaffolds particularly the one that contains 50% PLGA, 25% SF and 25% collagen is more suitable for nerve tissue engineering compared with PLGA nanofibrous scaffolds [108]. Besides blending electrospinning, surface conjugation also have been used. Collagen was immobilized onto the electrospun PMMAAA (copolymer of methyl methacrylate and acrylic acid) nanofiber surface. NSCs were cultured on the random nanofibers, and the results indicated that the cell behaviors, such as attachment and viability, were improved by the immobilized collagen [119].

Pure synthetic fibers could support nerve cell growth and proliferation, and the capability is limited partially because of its poor hydrophilicity. Plasma treatment is a simple and cost-effective way to improve the hydrophilicity of synthetic polymeric scaffolds. The hydrophilicity of PCL nanofibers was improved by plasma treatment, and the proliferation of SCs was significantly increased [98].

### Aligned nanofibers

For nerve tissue applications, it is important to guide the cells grow in a certain direction and facilitate the alignment of the nerve cells. Aligned nanofibers are more desirable than those random nanofibers on the point of benefiting neurite growth, which has already been highlighted in the previous article [41, 94]. The aligned nanofibers display appropriate capabilities of guiding neurite direction and cellular alignment. Many attempts have been made to fabricate aligned fibers and prove its efficiency in nerve tissue engineering. The normal way to prepare aligned fibers is to use a high-speed rotating drum as the collector, and different polymers have been successfully electrospun to be aligned nanofibers.

Neurites were directed down the axis of the highly aligned PLA fibers, while neurites also grew along the random fibers. At times, these random fibers even stopped further axonal extension. Highly aligned PLA fibers guided neurite and SC growth along the aligned fibers [93]. The effect of nanofibers alignment on nerve cells’ behavior was studied by culturing neurites from dorsal root ganglia (DRG) on PLA nanofibers with different alignments. Better alignment could guide the cell growth and benefit the neurite outgrowth with 20% and 16% longer than those on random and less aligned fibers [94]. Aligned poly(propylene carbonate) (PPC) nanofibers were fabricated, and the results indicated that the aligned PPC nanofibers instead of randomly oriented fibers significantly enhanced peripheral nerve regeneration *in vitro* [124]. Neurites of rat DRG displayed a directional growth that mimicked the fiber alignment on aligned polydioxanone nanofibers after 10 days culture [85]. Adult NSCs were cultured on aligned PCL/octadecylrhodamine B chloridenanofibers elongated along the major fiber axis, and a higher fraction of cells on aligned fibers exhibited markers of neuronal differentiation as compared with cells on random fiber on unpatterned surfaces [126]. PLGA aligned fibers were prepared, and the effect of nanofibrous structure on nerve cell directional proliferation and morphology has been studied. The results recommend that the best structure to promote cell direction, morphology and proliferation is accessible in an optimized hydrophilicity and porosity of scaffolds, which was obtained at the collector linear speed of 2.4 m/s [105].

Based on the successful application of abovementioned aligned nanofiber into nerve cell culture, the function of aligned nanofibers to support neuronal differentiation of stem cell and the in *vivo* researches were performed. Aligned PCL nanofibers could support the differentiation of ES cells into specific neural lineages. And aligned nanofiber substrates could discourage the differentiation of ES cells into astrocytes, which may limit the possible glia scar formation and facilitate the cure of spinal cord injuries. And aligned nanofibers are capable of directing and guiding neurite extension over significant distances [101]. Aligned PLGA fiber could guide cells properly orientated along the direction of the scaffold; and in animal studies, 8 weeks after the scaffolds were engrafted to bridge 3 mm defects of 10 adult rat spinal cords, locomotor and sensory scores of grafted animals were found to be significantly better than the control group. The scaffold supported the axonal regeneration of injured spinal cords and regenerating axons were seen to enter the graft and extend along its length [106].

Natural polymers such as collagen, gelatin and SF are frequently blended into the synthetic polymer scaffolds to improve the biocompatibility of the nerve scaffolds. For electrospun nanofibers, the addition of 25% collagen into PCL could enhance the biological effects of SCs, such as migration and neurite orientation. And analysis of isolated sensory neurons showed significantly better axonal guidance by the Collagen/PCL material [41]. Another research showed that cell adhesion and migration by hNP-AC (human neural progenitor-derived astrocytes) were clearly improved by functionalization of nanofiber surfaces with type I collagen (25%). Long axonal growth (up to 600 µm in length) of SH-SY5Y neurons followed the orientation of both types of nanofibers even though adhesion of the processes to the fibers was poor [99]. PLA-CL, Collagen I and Collagen III are utilized for the fabrication of aligned nanofibers by electrospinning. C17.2 cell proliferation on aligned PLA-CL/collagen I/collagen III scaffolds showed 22% increase compared with that on aligned pure PLA-CL scaffolds [113]. PCL/gelatin fibers were fabricated, and full alignment of fibers promoted nerve cell recognition and directed neurite outgrowth [100]. PLA-CL/SF fiber with the weight ratio of 25:75 were prepared by electrospinning method. Proliferation of SCs is significantly promoted, and the cell elongation is regulated to be aligned on aligned nanofibrous scaffolds [112].

### 3D nanofibers

Ultimate artificial nerve graft should be a 3D structure conduit, and it can be applied in bridging the nerve lesion. 3D nerve conduits based on electrospun nanofibers of diverse constitutes have been developed.

A design of a 3D scaffold consisting of parallel fibers embedded in a collagen matrix were developed. Using primary cultures of embryonic chicken DRGs, the results showed that PCL microfibers in the 3D matrix guide the direction of SC migration and axonal growth [104]. 3D electrospun PCL scaffolds modified with ethylenediamine (ED) were fabricated, and the interaction of rat brain-derived NSCs on the randomly orientated submicron PCL fibrous scaffolds was investigated. The modified scaffolds exhibited higher hydrophilicity, which resulted in a significant increase in the number of adhered cells and the enlargement of the cell spreading was observed within the entire scaffold. NSCs seeded on the PCL scaffolds in the presence of 10% FBS differentiated primarily into oligodendocytes, confirming the potential of electrospun PCL substrates to directed differentiation of NSCs toward specific tissues [102].

3D nerve scaffolds have already been used for *in vivo* study, fabricated either by single polymer or blending of different polymers. Poly (3-hydroxybutyrate-co-3-hydroxyvalerate) (PHBV) nanofibrous nerve conduit cross-linked by chitosan were used to treat a 10-mm gap in the sciatic nerves of the rats. The results showed that SCs were well attached on the surface, and especially in the nanofibrous graft, the sciatic nerve trunk had been reconstructed with restoration of nerve continuity and formatted nerve fibers with myelination [116]. PLGA/PCL blended electrospun tubes were used to regenerate a 10-mm sciatic nerve gap in rat. Four months after surgery, nervous regeneration and functional reconnection of the two severed sciatic nerve tracts are induced in most of the treated animals with the electrospun tubes. Myelination and collagen IV deposition, as well as regenerated fibers were observed, and at the same time no significant inflammation happened [103]. Electrospun PLGA–SF nerve conduit was employed to bridge 10 mm defects in the sciatic nerves of Sprague Dawley rats. Six weeks after the operation, morphological and functional assessment showed that nerve conduits from PLGA–SF grafts promoted the regeneration of peripheral nerves [107].

### Functionalized scaffolds for nerve TE

In addition to the topographic features, biofunction of scaffolds is also very important for nerve tissue regeneration. Functionalization of the nerve scaffolds normally can be gained by addition of biomolecules, including proteins and its related peptides. They have been incorporated into nanofibers, which offer new opportunities for the preparation of functionalized bioactive nerve scaffolds. Nervous system produces large protein molecules that regulate cell division, cell survival and neurite outgrowth. During the nervous system development, nerve target cells or surrounding glia secretes neurotrophins, and only the neurons that are receiving sufficient amounts of neurotrophins will survive. In addition to neurotrophins, ECM-based proteins and other molecules have been proved to be proficient for nerve tissue regeneration.

#### Neurotrophic factors

Neurotrophic factors are a family of proteins that play numerous roles in the nervous system including both PNS and CNS. With the assist of neurotrophic factors, nerve cell behaviors, such as viability, proliferation, differentiation, axonal outgrowth and apoptosis, are greatly enhanced [127]. Neurotropins includes neurotrophin-3 (NT-3), NGF, BDNF and neurotrophin-4/5 (NT-4/5) [128, 129]. Inconstant quantities of neurotrophins are produced in the brain, and they distribute in different regions [130]. Among these neurotrophins, NGFs play important role in the growth, maintenance and survival of nerve cells and support peripheral nerve regeneration in a rat model [36, 131]. Neuron survival and differentiation are greatly affected by BDNF, NT-3 and NGF in the PNS. During embryogenesis, cell proliferation, the differentiation of new neurons and synaptogenesis are supported by NT-3 [132].

Chitosan/poly(vinyl alcohol) (CS/PVA) conjugated NGF scaffolds were assessed in terms of attachment and proliferation of SKNMC and U373 cell lines. Cytocompatibility, cell viability and preliminary bioactivity assays have given important evidence that all systems evaluated are nontoxic, bio-tolerant and potentially biocompatible [122]. The growth of neurites increased 2-fold on aligned SF fibers containing BDNF, 2.5-fold on aligned SF fibers containing CNTF and by almost 3-fold on aligned SF fibers containing both factors [121]. NT-3 encapsulated electrospun collagen scaffolds enhanced nerve cell behaviors and neurite outgrowth, and the growth factor encapsulating scaffolds are promising for SCI treatment with satisfactory morphological properties and biochemical cues that can facilitate nerve regeneration [125].

A comprehensive combination of several advanced methods such as aligned nanofibers, core-shell structure and bioactive growth factors have been applied to improve the efficiency of nerve tissue regeneration, and satisfactory results have been gained which was proved by the *in vivo* study. Nerve guidance conduits (NGCs) consisting of aligned SF/PLA-CL nanofibers encapsulating NGF which presented a sustained release and remained biological activity over 60 days was used as a bridge implanted in the sciatic nerve of rats, and it was able to promote nerve regeneration [115, 133]. NGCs consisting of aligned NGF incorporated core-shell PLAG nanofibers was used to treat a 13-mm rat sciatic nerve defect. The results indicated that the functional recovery of the regenerated nerve in the PLGA/NGF group was significantly better than that in the PLGA group, yet had no significant difference compared with the autograft group [110].

#### ECM proteins and their sequences

ECM, which typically consists of fibronectin (FN), laminin (LM), collagen, tenascin and thrombospondin, greatly affects cellular activity. Laminin, tenascin and thrombospondin are beneficial for cell proliferation [134], while laminin and collagens are able to regulate the differentiation of neural precursor cells and influence cell adhesion and neurite growth [135]. Collagen is a widely used natural polymer for preparation of nerve scaffolds, and the related research have been introduced in the above parts of this article. One of its most important advantages is the good electrospinability, while pure laminin and fibronectin are not electrospinable.

Laminin is a heterotrimeric glycoprotein consisting of α, β and γ subunits [136]. As a major component of basement membrane of ECM, laminin has been undergoing several investigations both *in vitro* and *in vivo* on its influence on neurite outhgrowth [137, 138]. Potential of laminin to mediate cell survival, axon extension and cell adhesion through specific peptide sequences, along with importance of its role in integrin signaling on nerve tissue regeneration has been well studied [139]. Laminin is often combined with other supportive materials to enhance growth promotion and cell differentiation because it is not electrospinable [140]. The efficiency of aligned fibers were discussed in above parts. Based on aligned nanofibers, aligned and bioactive nanofibers were developed by immobilizing ECM protein laminin and growth factor (FGF) on nanofibers, which significantly induced neurite outgrowth and promoted highly efficient neurite outgrowth [95]. Core-shell structured nanofibers can further enhance the laminin function on SCs. Sustained release of laminin from core-shell nanofibers could support anchoring and proliferation of SCs; after 7 days *in vitro* culture, the proliferation of SCs showed 78% increase on core-shell nanofibers than that on blending nanofibers. Additionally, the cells expressed bipolar and tripolar elongations [111].

Fibronectin is a high molecular weight glycoprotein that binds to integrins and other ECM proteins such as the collagen [141]. It is also native to nerve architecture, such that it assist towards the outgrowth of neurites [142]*.* Although fibronectin is incapable of forming a hydrogel or other scaffolds on its own, it has been used in combination with hydrogels for spinal cord repair due to its cell-signaling properties through RGD–integrin binding [143].

Instead of the proteins, peptides RGD, IKVAV and YIGSR, isolated from fibronectin or laminin, are preferred to improve the biological activities of nerve conduit [144]. A bilayered chitosan tube that comprises an outer layer of chitosan film and an inner layer of chitosan electrospun nanofibers combined with YIGSR peptide were grafted to bridge injured sciatic nerve, and has been shown to promote nerve repair in rats [123]. Functionalized electrospun PCL fibers with biologically active peptides GRGDS, which derived from ECM proteins, were fabricated. A marked effect of GRGDS functionalization on proliferation of the SC, which more than tripled in comparison to PCL fibers not carrying the peptide was observed [97].

More comprehensible understanding of protein-mediated material–cell interaction paired with use of those electrospun particles may become new approach in neural tissue engineering.

#### Other molecules

In addition to the frequently used neurotrophins, ECM-based protein and its peptides, some other molecules also have been used for nerve tissue engineering in recent years. After CNI, formation of glial scar is the greatest barrier for recovery and regeneration. Glial scar produces some inhibitory factors, such as chondroitin sulfate proteoglycans (CSPGs) [145]. The general idea is to deliver some factors which can digest the CSPGs, which will help create the environment less inhibitory for the regeneration. And delivery of chondroitinase ABC (ChABC), which can digest the side chain of CSPGs, has been attempted with some success [146, 147].

### Advanced regeneration of the nerve

#### Conductive biomaterials and electrical stimulation

In addition to the imitation of native nerve structure and the biomolecule components, research on conductive polymers and electrical stimulation emerge as a relatively novel approach to increase neurite extension and axonal regeneration [148, 149]. Barriers and cell membranes form ionic barriers, resulting in an electrical potential gradient developing across its surface, and stimulate nerve growth toward the periphery [150]. This led to the application of conductive biomaterials which are able to enhance neurite extension with low electrical stimulation, such as polypyrrole (PPy) and polyaniline (PANi).

Aligned PLGA nanofiber coated with PPy supported the growth and differentiation of rat PC12 cells and hippocampal neurons comparable to noncoated PLGA control meshes. PC12 cells, stimulated with a potential of 10 mV/cm on PPy-PLGA scaffolds, exhibited 40–50% longer neurites and 40–90% more neurite formation compared with unstimulated cells on the same scaffolds [109]. Neurite extension area was increased when photoresist patterns were doped with electrically conductive polymers, PPy as well as conjugated NGF and poly-l-lysine/laminin [151, 152]. Nerve stem cells cultured on blended PLLA/PANi (85:15) scaffolds exhibited extended neurite outgrowth after 60 min of *in vitro* electrical stimulation using electric field of 100 mV/mm, which was not observed for cells cultured on nonstimulated scaffolds. PLLA-PANi scaffolds could direct NSC differentiation, as electrical stimulation of PLLA-PANi scaffolds promoted elongated, neurite morphology of NSCs compared with unstimulated controls [92]. PC12 cell viability was significantly higher on blended PLA-CL/PANi fibers than on pure PLA-CL fibers, and PC12 cells cultured on PANi containing fibers expressed more specific proteins, and additionally the cell body grows in aligned direction with outgrowing neurites. The results showed that neuronal differentiation of PC12 cell is greatly affected by the conductivity of the substrates [114].

#### Cells for regeneration of the nerve

Therapies involving stem cells have showed the potential in curing the tissue injury, and controlling of cell distribution and viability are the two key technological points. Improved viability, neurite outgrowth and neural function recovery were gained with the help of collagen scaffold enhanced cell therapy [153]. And also NSC and SC loaded PLGA scaffolds supported axonal regeneration in the transected spinal cord, and significantly more axons in the NSC and SC treated groups compared with the control group [154]. So a combination of electrospun fiber scaffolds, NSCs and controlled delivery of instructive cues could lead to the development of a better strategy for nerve injury repair.

Binan *et al.* developed a nonwoven material made of coaxial electrospun fiber of PLA and gelatin with a degradation rate and mechanical properties similar to peripheral nerve tissue, and investigated their effect on cell survival and differentiation into motor neuronal lineages through the controlled release of retinoic acid (RA) and purmorphamine. Engineered neural stem-like cells (NSLCs) seeded on these fibers, with and without the drugs, differentiated into β-III-tubulin, HB-9, Islet-1 and choactase-positive motor neurons by immunostaining in response to the release of the biomolecules. In addition, the bioactive material not only enhanced the differentiation into motor neuronal lineages but also promoted neurite outgrowth [96]. Conjugated NGF to nanofibers could enhance the expression of neuronal differentiation markers for MSCs after 5 days culture [118].

### Electrosprayed micro/nanoparticles and the potential in nerve tissue engineering

With the same equipment to electrospinning, micro/nanoparticles also can be gained by the process ‘electrospraying’ by simply adjusting the solution properties or process parameters. Micro/nanoparticles are ideal drug carrier, which can further be applied in the target injured organ by injections instead of invasive surgery. In contrast to other encapsulating methods, electrospraying is single-stage process which allows to obtain higher load efficiency and narrower particle size distribution [155].

**Figure 4.**
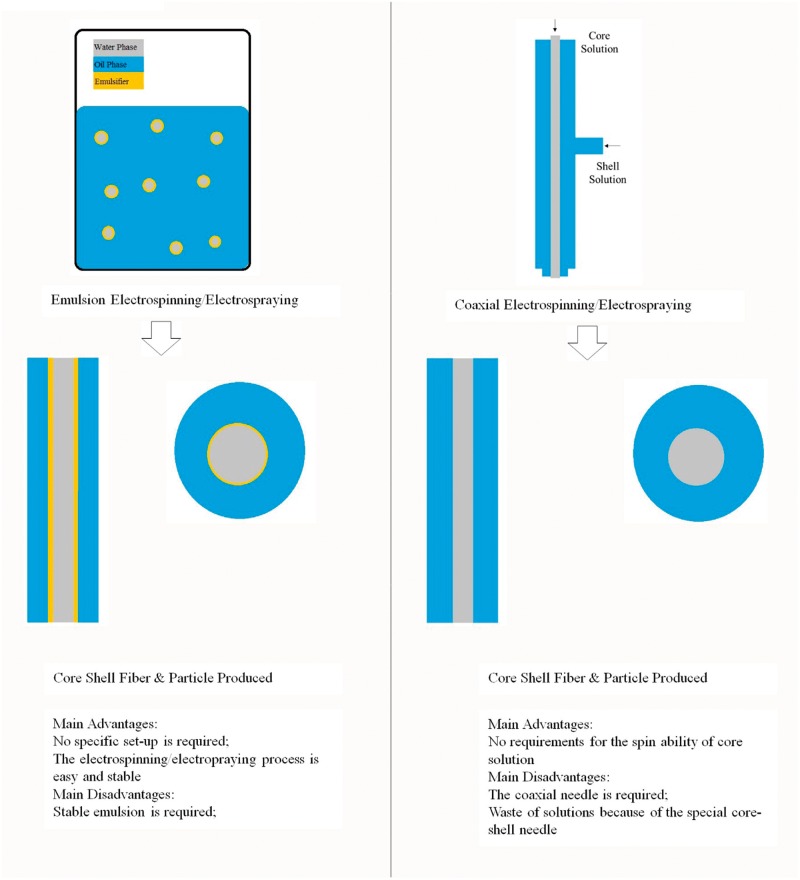
Comparison between emulsion and coaxial electrospinning and electrospraying.

Electrospray could be conducted in different ways which have been applied into electrospinning, such as blending electrospraying, coaxial electrospraying and emulsion electrospraying. Blending of the drugs in a polymer solution is the simplest way for drug and even for cells encapsulation into the electrosprayed particles, and during which adequate physical interactions between the polymer and the drugs are crucial for the release behavior [155]. Blending would lead to problems of initial burst release, especially when the drugs and polymers are not compatible [156]. Coaxial electrospray using coaxial needles, which does not require the compatibility of core and shell solutions, could be a solution, and thus the drugs can be encapsulated in the core part of the particles. Xie *et al.* encapsulated bovine serum albumin (BSA) and lysozyme in the microparticles by coaxial electrospray, and the *in vitro* release profiles indicated that sustained release of proteins for more than 30 days [157]. In addition to coaxial electrospraying, emulsion electrospraying should be another easy way to produce micro/nanoparticles with core-shell structure. Emulsions have been proved to be stable during electrospraying and a fine stable jet spray could be produced [158]. Dual drugs loaded core-shell nanoparticles have been fabricated, and it was noted that a programmable release pattern for dual drugs was also achieved by adjusting the loading regions in the core-shell structures [159]. PLGA microparticles with incorporated protein via either emulsion or coaxial electrospray techniques have been done, during which PLGA was used as the carrier and BSA as a model protein. The results show that the coaxial electrospray microparticles presented a much slighter release than the emulsion electrospray microparticles, and loading efficiency was significantly higher (*P* < 0.05) in the coaxial group than emulsion group [160]. The comparison between emulsion and coaxial electrospinning and electrospraying is shown as in Fig. 4.

Using the diverse forms of electrospraying, bioactive drugs have been encapsulated inside the particles. And BSA was encapsulated in various polymeric matrices by electrospraying. BSA loaded PLGA microparticles were achieved from electrospraying process, and the BSA could maintain integrity, and as well as the secondary structure after release from polymeric microparticles [161]. BSA was encapsulated inside electrosprayed tripolyphosphate (TPP) cross-linked chitosan capsules. Electrospraying parameters, such as the solution concentration, flow rate and ratios of the constituents have a greater influence on the morphology, loading efficiency and *in vitro* release of BSA/chitosan particles, and this has been researched comprehensively by Xu et al. [162]. Water in oil emulsion was electrosprayed for preparation of BSA loaded particles [163]. Protein drug encapsulation like vaccines, growth factors or signaling proteins have been researched, and the results show that the loaded proteins are slowly released to the body fluids. Insulin also has been entrapped into the lipid particles with high encapsulation efficiency using electrospraying [164].

Other than proteins such as BSA, cells also have been successfully encapsulated into the microparticles using electrospraying. Microencapsulation of living cells (hepatocytes G2) in calcium alginate with controllable size and narrow size distribution, for their protection from immune system, was achieved using electrospraying. The obtained beads were of uniform size from 200 to 340 µm [165].

For nerve tissue engineering, especially for the brain, injection of drug-encapsulated micro/nanoparticles could be a promising therapy for TBI or neurodegeneration disease. This therapy could overcome the brain–blood barrier, and as well as reduce the risk of craniotomy.

## Conclusions and Perspectives

Current techniques and strategies that may allow for neuronal tissue regeneration or replacement, which is mainly related to electrospinning techniques, were reviewed. Both in the PNS and CNS, manipulation of the natural regenerative ability of the host could significantly enhance the reconstruction of severed or damaged neural tissue. Electrospun nerve scaffolds in diverse forms of aligned nanofibers, 3D conduit, core-shell fibers within growth factors in the core have been studied for years. A variety of polymers including synthetic polymers such as PLA, PCL, PLGA, PLA-CL, PHBV and natural polymers such as collagen, gelatin and SF, as well as neurotrophins, ECM-based proteins and other molecules have been applied for electrospinning, and the efficiency for improving the nerve regeneration have been proved.

But presently there still is a certain gap between electrospun biomaterials for nerve repair and the clinical standard of care, especially for SCI and TBI. Response to injury or disease vary greatly between CNS and PNS due to the different structural properties and cellular components, which requires exclusive therapy for individual applications, especially for CNS injury. It is convinced that the advanced nerve scaffold of the combination of electrospun biomaterial structure, bioactive molecules and the stem cells in a smart and organized way could play its role in nerve regeneration for both traumatic PNI and central nerve injury, even as well as for neurodegeneration diseases. Other than electrospun nanofibrous scaffolds, electrosprayed micro/nanoparticles could be a very promising drug carrier which could be used to cure TBI and neurodegeneration disease by injection instead of craniotomy. But the feasibility and efficiency need to be proved by future researches.
